# Supplementary material for: Blood pH Analysis in Combination with Molecular Medical Tools in Relation to COVID-19 Symptoms
Source: Biomedicines. 2023 May 11;11(5):1421. doi: 10.3390/biomedicines11051421 (PMC10216059; doi:10.3390/biomedicines11051421)
Supplement: Supplementary file 1 [file biomedicines-11-01421-s001.zip › biomedicines-2294535-supplementary.pdf]

Supplementary Figures: H-C Siebert *et al*.: Blood pH Analysis in Combination with Molecular Medical Tools in Relation to Long Covid Symptoms

(***Figure S1***)

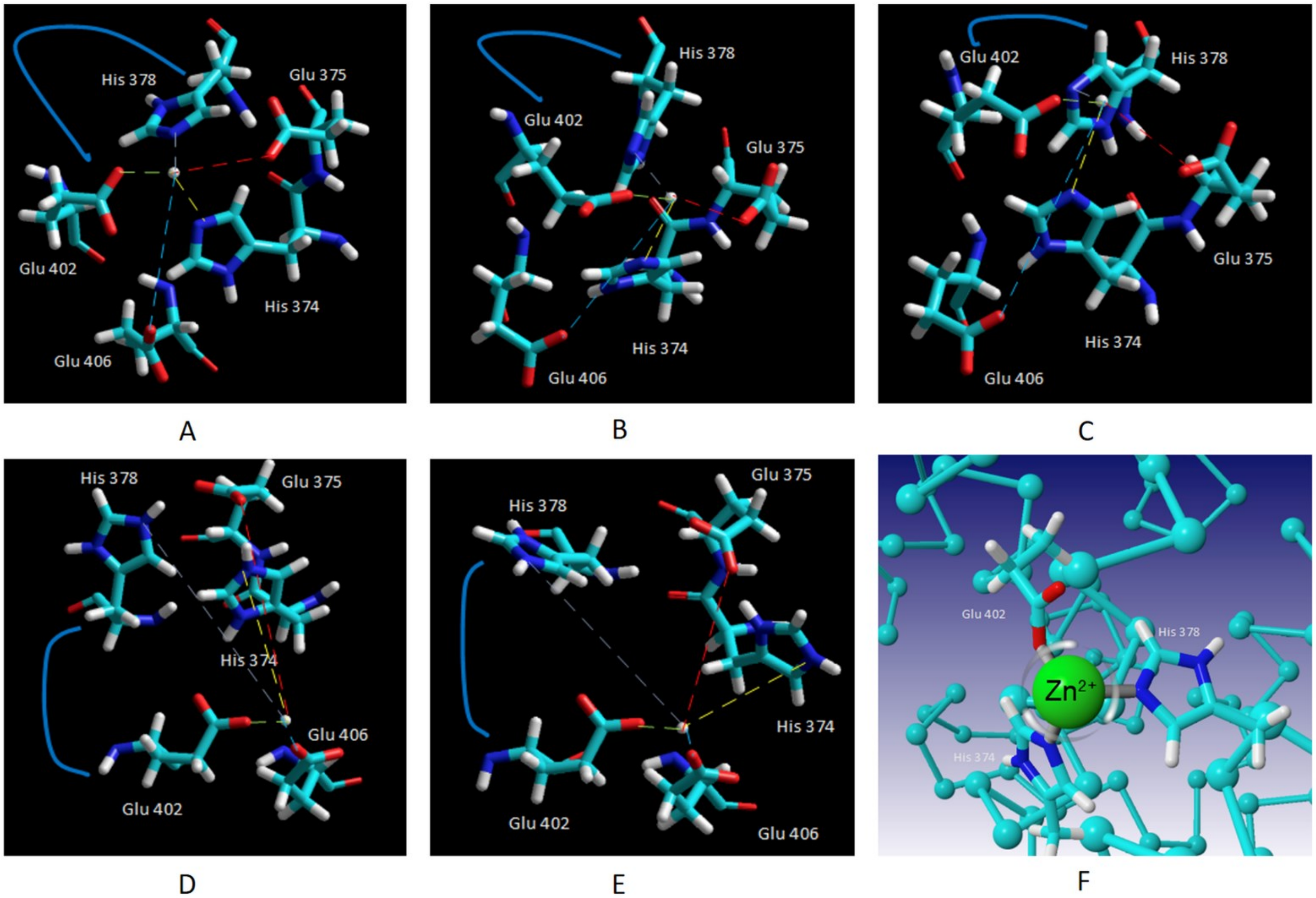

Figure S1. The pH-dependence of the ACE2 receptor in complex with the SARS CoV2 spike protein as revealed by molecular dynamics (MD) simulations. **A)** Crystal structure (6M0J.pdb) of the Zn^{2+}-related fragment used as input for MD simulations; **B)** pH 6.4; **C)** pH 6.2; **D)** pH 6.0; **E)** pH 5.8; **F)** Magnified view of the Zn^{2+} environment.

(***Figure S2***)

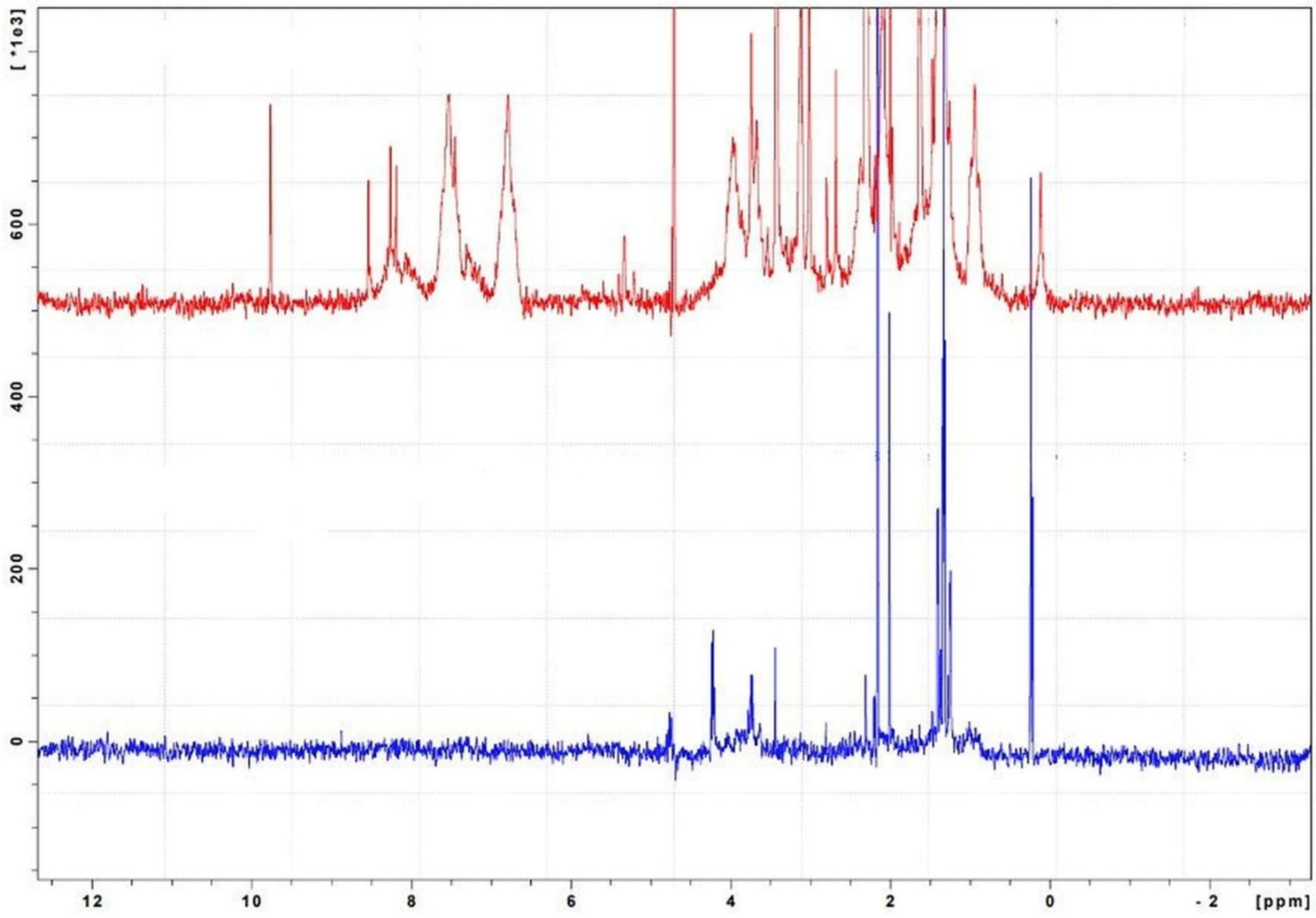

Figure S2. 1D proton (1H) NMR spectra of Pt2His (top, red) and Semaglutide (bottom, blue). Spectra recorded at 42°C in H2O (buffered at pH 7,4). Under these conditions the resolution of Pt2His is poor and the resolution of Semaglutide is extremely poor. A suitable encapsulation materials can be used to improve the resolution of the NMR signals.

(*Figure S3*)

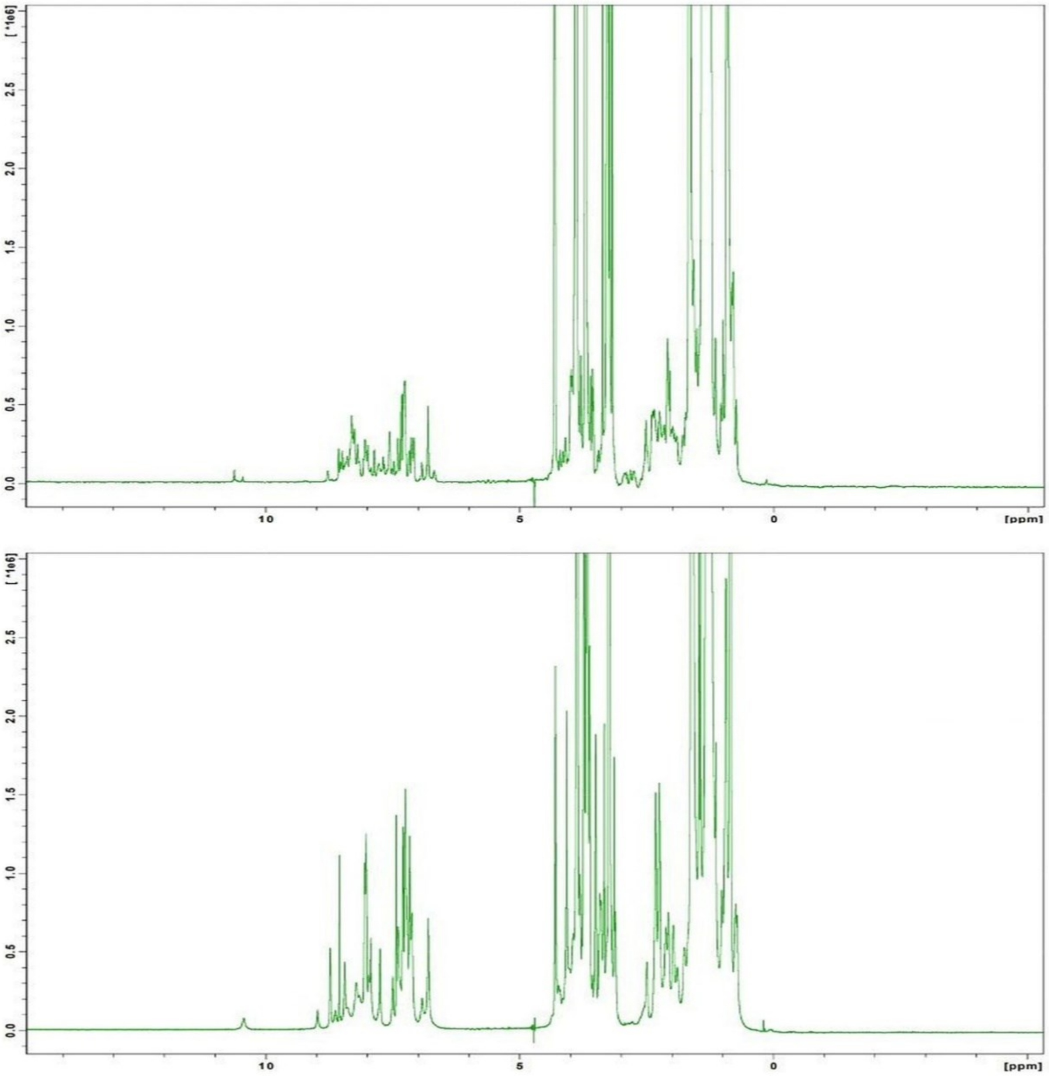

Figure S3. 1D proton (1H) NMR spectra of Pt2His (top) and Semaglutide (bottom) recorded at 37°C in the presence of mixed micelles consisting of DPC (dodecylphospho-choline) and the ganglioside GM1.

(***Figure S4***)

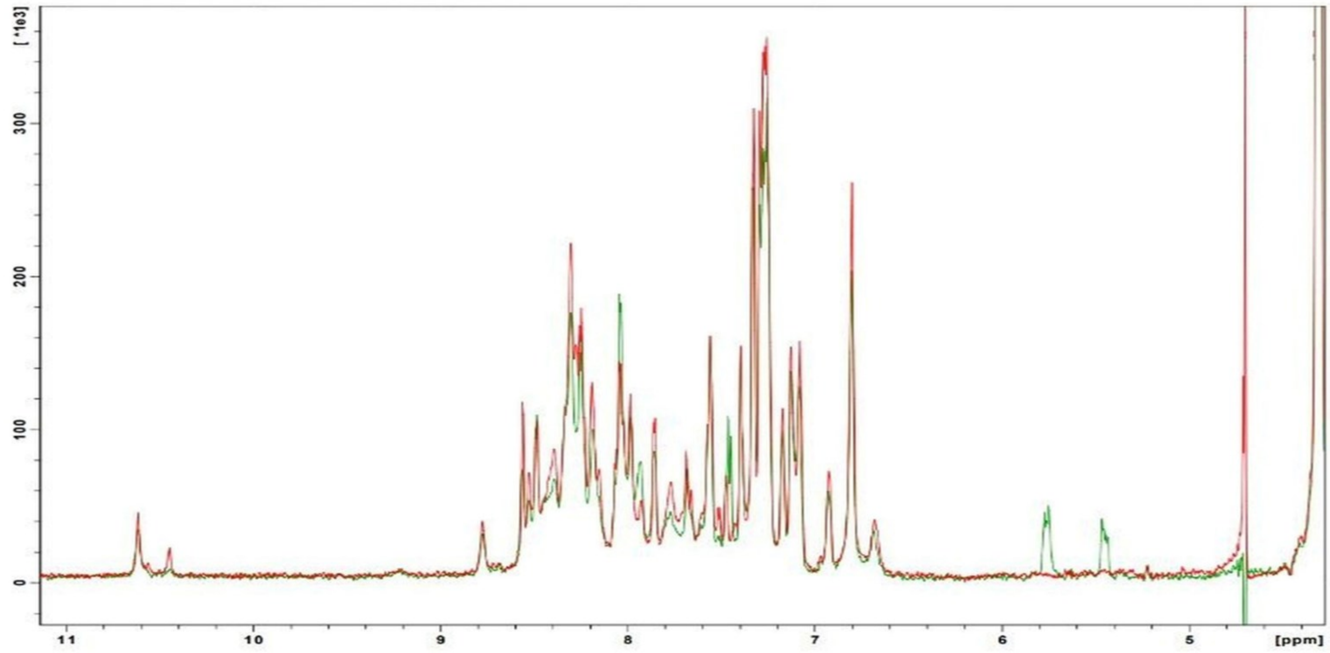

Figure S4. NH- and aromatic part of an 1D proton (1H) NMR spectra of Pt2His recorded at 37°C in the presence of micelles consisting of DPC (red) and mixed micelles consisting of DPC and the ganglioside GM1 (green).

(***Figure S5***)

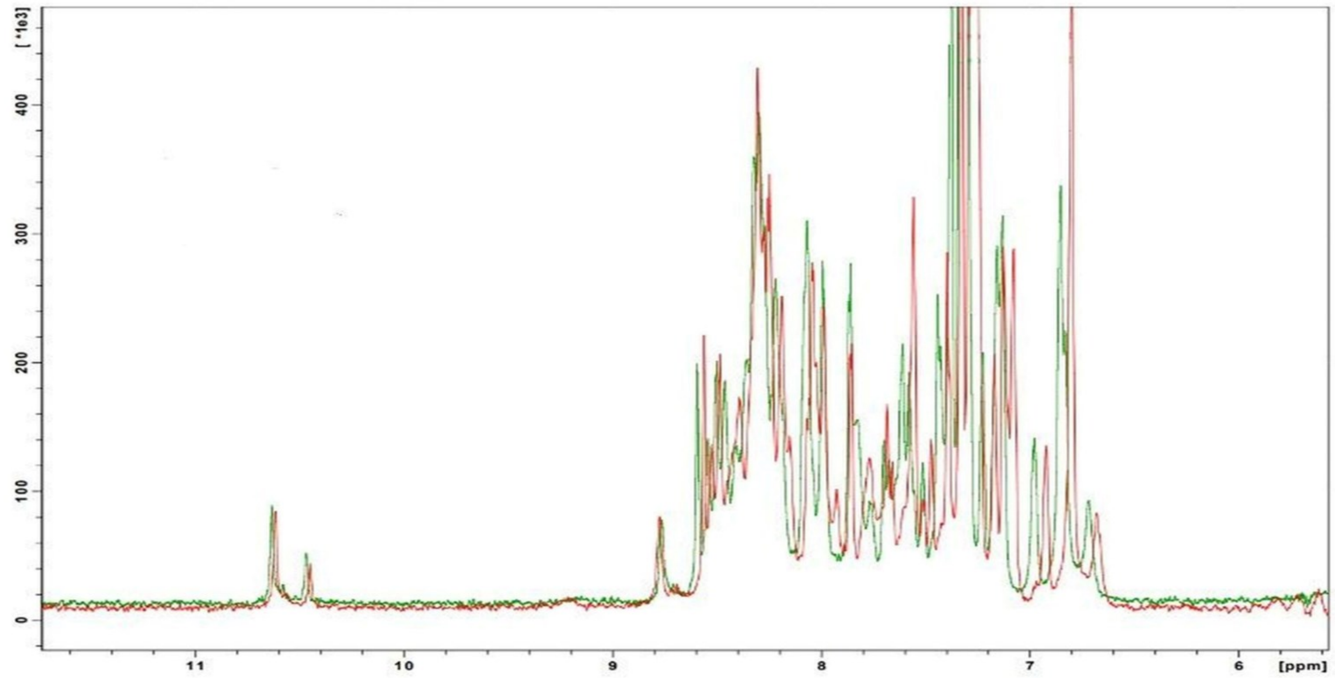

Figure S5. NH- and aromatic part of an 1D proton (1H) NMR spectra of Pt2His in the presence of mixed micelles consisting of DPC and the ganglioside GM1 recorded at 37°C (red) and at 42°C (green).

(**Figure S6**)

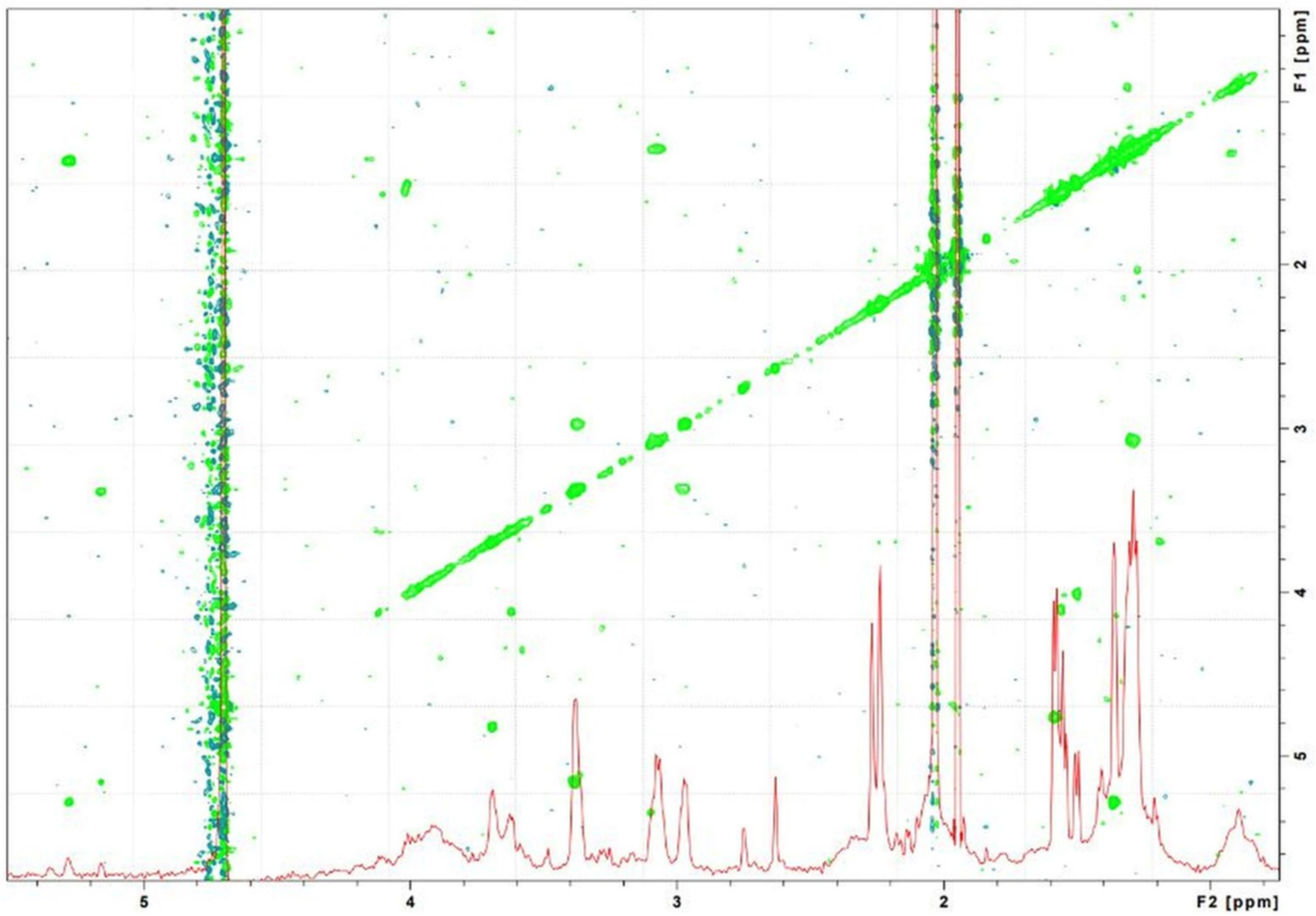

Figure S6. *Pt2His in H2O (buffered at pH 7,4) and recorded at a measurement time of 42°C. NH- and aromatic part of a 2D 1H TOCSY NMR spectrum combined with a 1D 1H NMR spectrum. The poor resolution of Pt2His without DPC leads to a 2D 1H TOCSY NMR spectrum with poor resolution.*

(***Figure S7***)

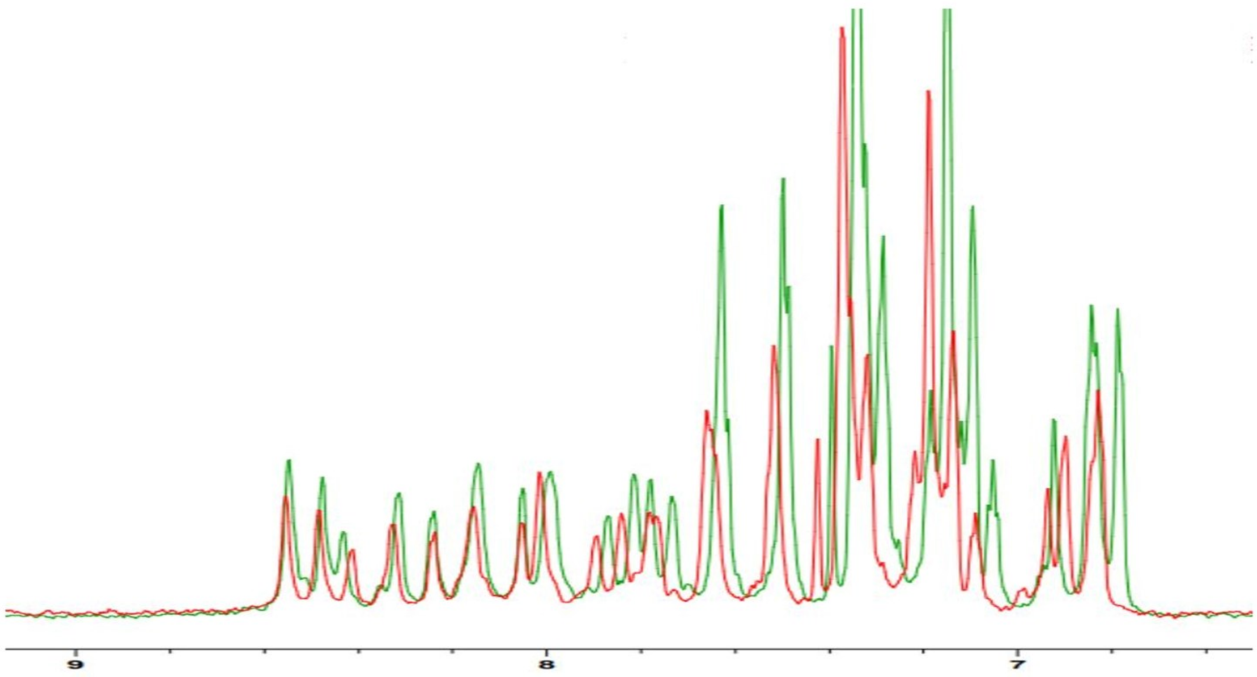

Figure S7. NH- and aromatic part of an 1D proton (1H) NMR spectrum of 2RUM in the presence of DPC and GM1 at 37°C (green) and at 42°C (red). NH and the aromatic part (left side) of a 1D (one-dimensional) proton NMR spectrum. The molar ratio of the SARS CoV fusion peptide 2 RUO as well as the ganglioside GM1 to DPC was 1:40.

(**Figure S8**)

A

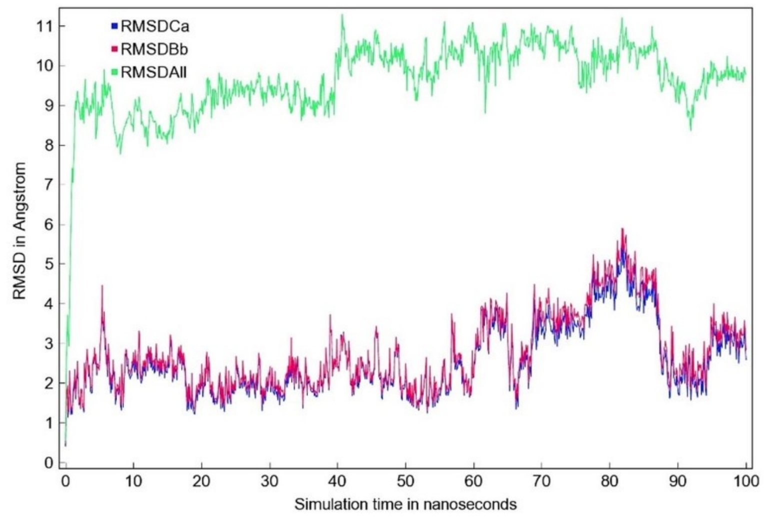

B

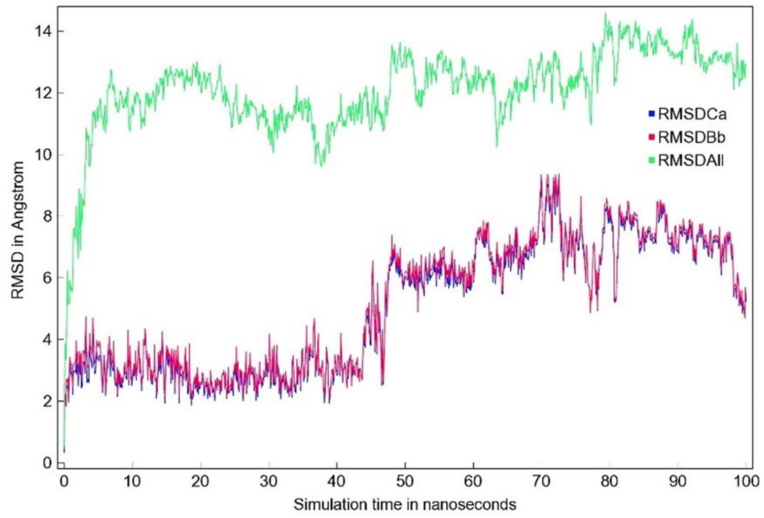

C

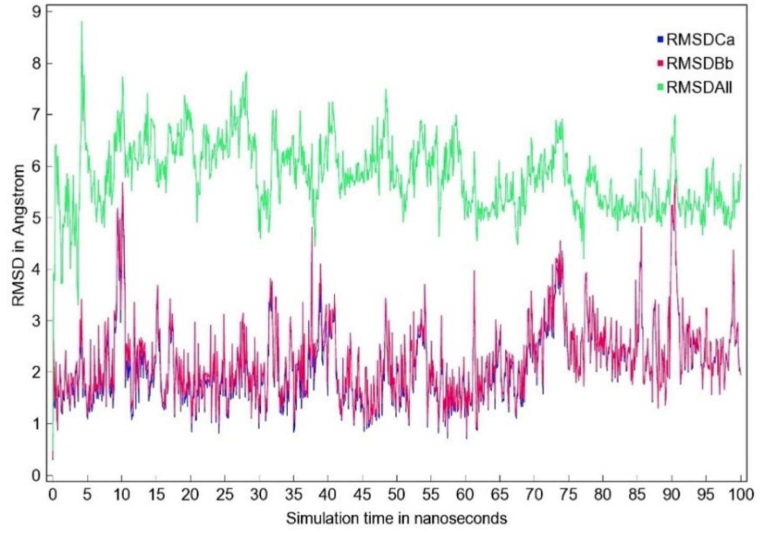

Figure S8. MD simulations of Tirzepatide, Semaglutide and Pt2His were conducted to assess the structural stability of the incretin-mimetics. In order to analyze the MD simulations, the RMSD was determined as a function of 100 ns running time (Figure S8 A, B, C). Figure S8 A shows an average RMSD of about 2 Å. Higher RMSD values were adopted when the fatty acid residue wrapped around the peptide.

(Figure S9)

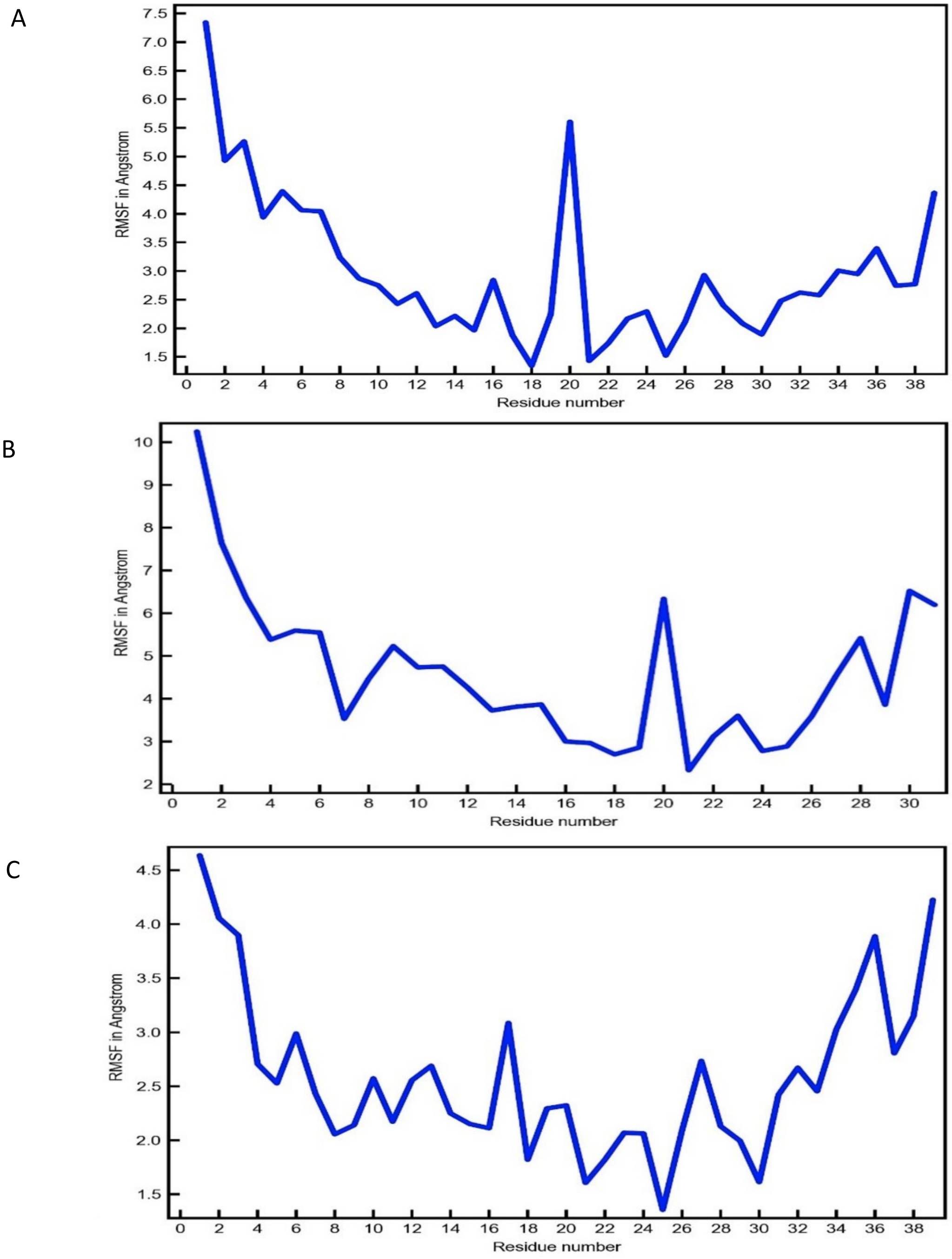

Figure S9. In order to analyze the origin of the structural fluctuations, RMSF values for the individual residues were calculated (Figure S9 A, B, C). In particular, the ends of the peptides are very flexible. Furthermore, the amino acid Lys (Res 20) in Tirzepatide and Semaglutide has an increased flexibility due to the connection of the peptide chain to the fatty acid residue at this point. Pt2His (Figure 8 C) also shows an RMSD value around 2 Å. Higher RMSD values exist when the fatty acid residue wraps around the peptide. The fatty acid residue in Pt2His is linked to Lys 14. Interestingly, the RMSF of Lys 14 is not particularly high. A strong increase in the RMSF (Figure S9 C) can be seen at Arg 17, which the MD simulation indicates an increased interaction with the fatty acid side chain.
